# Investigation of the Matrix Metalloproteinase-2 Gene in Patients with Non-Syndromic Mitral Valve Prolapse

**DOI:** 10.3390/jcdd2030176

**Published:** 2015-07-10

**Authors:** Maëlle Perrocheau, Soto Romuald Kiando, Déwi Vernerey, Christian Dina, Pilar Galan, Albert Hagege, Xavier Jeunemaitre, Nabila Bouatia-Naji

**Affiliations:** 1 Paris Cardiovascular Research Center, INSERM UMR970, 56 rue Leblanc, Paris F-75015, France; E-Mails: maelle.perrocheau@gmail.com (M.P.); romuald.kiando@inserm.fr (S.R.K.); dvernerey@chu-besancon.fr (D.V.); albert.hagege@aphp.fr (A.H.); xavier.jeunemaitre@inserm.fr (X.J.); 2 Paris Descartes University, Sorbonne Paris Cité, 12 rue de l’école de medicine, Paris F-75006, France; 3 INSERM UMR1087, CNRS UMR 6291, Institut du Thorax, 8 Quai Moncousu, Nantes F-44007, France; E-Mail: Christian.Dina@univ-nantes.fr; 4 Centre Hospitalier Universitaire (CHU) Nantes, Université de Nantes, 8 Quai Moncousu, Nantes F-44007, France; 5 Equipe de Recherche en Epidémiologie Nutritionnelle (EREN), Centre d’Epidémiologie et Statistiques Sorbonne Paris Cité, Inserm (U1153), Inra (U1125), Cnam, Université Paris 13, COMUE Sorbonne Paris Cité, Bobigny F-93017, France; E-Mail: p.galan@uren.smbh.univ-paris13.fr; 6 Department of Cardiology, Hôpital Européen Georges Pompidou, Assistance publique-Hôpitaux de Paris (AP-HP), 20 rue Leblanc, Paris F-75015, France; 7 Department of genetics, Hôpital Européen Georges Pompidou, Assistance publique-Hôpitaux de Paris (AP-HP), 20 rue Leblanc, Paris F-75015, France

**Keywords:** *MMP2*, mitral valve prolapse, Single Nucelotide Polymorphism, genetic association

## Abstract

Non-syndromic mitral valve prolapse (MVP) is a common degenerative valvulopathy, predisposing to arrhythmia and sudden death. The etiology of MVP is suspected to be under genetic control, as supported by familial cases and its manifestation in genetic syndrome (e.g., Marfan syndrome). One candidate etiological mechanism is a perturbation of the extracellular matrix (ECM) remodeling of the valve. To test this hypothesis, we assessed the role of genetic variants in the matrix metalloproteinase 2 gene (*MMP2*) known to regulate the ECM turnover by direct degradation of proteins and for which transgenic mice develop MVP. Direct sequencing of exons of *MMP2* in 47 unrelated patients and segregation analyses in families did not reveal any causative mutation. We studied eight common single nucleotide polymorphisms (TagSNPs), which summarize the genetic information at the *MMP2* locus. The association study in two case controls sets (N_Cases_ = 1073 and N_Controls_ = 1635) provided suggestive evidence for the association of rs1556888 located downstream *MMP2* with the risk of MVP, especially in patients with the fibroelastic defiency form. Our study does not support the contribution of *MMP2* rare variation in the etiology to MVP in humans, though further genetic and molecular investigation is required to confirm our current suggestive association of one common variant.

## 1. Introduction

Mitral valve insufficiency is a major cause of morbidity and mortality worldwide [1]. One of the main mechanisms contributing to mitral insufficiency is mitral valve prolapse (MVP), characterized by the systolic displacement or billowing of the mitral leaflets into the left atrium. MVP is the clinical expression of degeneration of the mitral valve. This causes the displacement of one or both enlarged, thickened mitral leaflets into the left atrium during systole [2]. MVP results from the consequence of a degenerative process with variable morphological presentations such as billowing mitral leaflets with excess of connective tissue and myxomatous degeneration, also named Barlow disease or thin elongated leaflets and chordae with fibroelastic and acid mucopolysaccharide deficiency [3]. Many patients with MVP show few clinical symptoms, if any. Nonetheless, MVP is the most common cause of isolated mitral regurgitation requiring surgical repair and a confirmed risk factor of heart failure, arrhythmia, endocarditis, and sudden cardiac death [1].

The etiology of MVP is complex and this includes acquired (e.g., rheumatic, trauma and ischemic origin) and genetic causes, including several connective tissue genetic disorders (e.g., Marfan and Elhers-Danlos syndromes) [4]. Several efforts have attempted to decipher the genetics of MVP using linkage within families in the past two decades. We have previously reported linkage studies of large families where four loci were linked to MVP on Chr16p11-12, Chr11p15.4, Chr13q31-32 and ChrXq28 [5,6,7,8]. Mutations in the FLNA, the filamin A gene, were demonstrated to cause the X-linked myxomatous MVP [9], which is to date, the only known causative gene for rare familial MVP. Filamin A regulates reorganization of the actin cytoskeleton and is essential for matrix organization during fetal valve development [10]. On the other hand, MVP is also present in several rare connective tissue syndromes, (e.g., Marfan, Ehlers-Danlos) [11], which supports a role of the extracellular matrix biology and cytoskeleton cell cross talk in valve development and integrity. At the population level, MVP prevalence is estimated ~3% [12] and thus presents a putative complex genetic pattern of inheritance where common genetic variants could be at play, especially in candidate genes and pathway.

The physiological function of metalloproteinases (MMPs) is the modulation and the regulation of extracellular matrix (ECM) turnover by direct proteolytic degradation of the ECM proteins, including collagen, fibronectin, and proteoglycans [13]. Previous studies suggested a role for the *MMP2* in the pathogenesis of MVP. In humans, a higher level of *MMP2* expression is reported in myxomatous valves compared to normal valves [14]. On the other hand, there is evidence for increased activity of *MMP2* in myxomatous valves compared to normal valves [15] and an ability *MMP2* to cleave collagen, elastin, and fibrillin [13,16]. In addition, although normal when young, 12–14 months mice with cardiac-specific constitutively active *MMP2* reproduce many of the features of the human MVP syndrome including mitral valve thickening and prolapse with severe myxomatous changes [17]. These data suggest the *MMP2* coding gene (*MMP2*) as a strong candidate gene for non-syndromic MVP.

The aim of this study is to investigate the link between genetic variants in *MMP2* and mitral valve prolapse, including mostly severe cases with mitral repair by surgery and Barlow, myxomatous disease. We first screened by direct sequencing all coding exons of *MMP2* in MVP patients and investigated the role of rare coding variants in genetic segregation in families. We have also performed a locus-based case-control association study to assess the role of common single nucleotide polymorphisms (SNPs) in and around *MMP2* in MVP genetic susceptibility in a case control study design. We have also performed stratified analysis in surgery cases and Barlow and fibroelastic deficiency-only forms of MVP.

## 2. Methods

### 2.1. Study Populations

#### 2.1.1. Familial MVP Index Cases

Sequencing of the *MMP2* coding regions was performed on DNA from 47 index cases selected from families affected by mitral valve prolapse recruited at the Department of Cardiology and the Department of Genetics of the “Hôpital Européen Georges Pompidou: HEGP”. Familial cases were defined as such when at least one additional member was affected in the family. The diagnosis of index cases (Familial cases, see Table 1) and relatives was determined by echocardiographic examination as previously described [5]. Patients were 54.4 years old on average; mostly men and 35% had already had surgery for valve repair or replacement (Table 1).

#### 2.1.2. MVP-France Cases

The MVP-France study (Genetic polymorphisms in idiopathic mitral valve prolapse: A French prospective study) is a prospective multicenter nation-wide study promoted by the French Society of Cardiology. Adult (≥18 years) patients with idiopathic MVP were included after written informed consent if they presented either: (1) leaflet prolapse defined as the displacement within the left atrium of any part of the mitral valve leaflet(s) ≥ 2 mm beyond a line connecting the annular hinge points on the parasternal long-axis view of the left ventricle by two-dimensional (2D) echocardiography [18]; and either increased leaflet thickness ≥ 4 mm measured in diastole at the mid-portion of the leaflet or mitral regurgitation ≥ 2+ based on extension of the regurgitant jet within the left atrium on a scale of 1 to 4+ using color Doppler; or (2) previous surgery for pure severe mitral regurgitation due to MVP supported by an operative report and written confirmation of the diagnosis by the surgeon.

**Table 1:** Characteristics of mitral valve prolapse populations and controls.

Cohort	*n*	Age at Examination (Years)	Sex Ratio (F%)	BMI (kg/m^2^)	Surgery (%)
Familial Cases	47	54.4 ± 15.6	45%	22.7 ± 2.7	35%
MVP-France (Set1)	625	62.7 ± 13.1	30%	24.3 ± 3.6	67%
Myxomatous (Barlow)	414	60.7 ± 13.5	30%	23.7 ± 3.2	64%
Non myxomatous (FED)	116	67.7 ± 10.7	33%	25.5 ± 4.1	67%
Surgery	421	63.7 ± 12.1	25%	24.5 ± 3.7	100%
MVP-France (Set2)	401	63.8 ± 12.2	26%	24.3 ± 3.7	80%
Myxomatous (Barlow)	255	62.5 ± 12.5	25%	23.9 ± 3.6	77%
Non myxomatous (FED)	57	67.6 ± 9.4	37%	25.6 ± 4.3	72%
Surgery	322	64.2 ± 11.8	24%	24.4 ± 3.6	100%
Controls					
SU.VI.MAX (Set 1)	815	65.8 ± 6.3	60%	NA	NA
SU.VI.MAX (Set 2)	820	67.3 ± 6.1	61%	NA	NA

FED: Fibroelastic deficiency form; NA: Not available.

An echocardiographic core laboratory (AH) validated echo cardiac recordings and operative reports. MVP-France recruitment excluded patients with MVP associated to heart disease (coronary artery disease, hypertrophic cardiomyopathy, or rheumatic disease) or syndromes (e.g., Marfan and Ehlers-Danlos). Clinical data were collected on electronic case report forms. Genetic core laboratory (XJ) centralized the collection of blood samples. Genomic DNA was successfully extracted from peripheral blood lymphocytes of patients using a commercial isolation kit and following the provider’s procedure (Qiagen^®^, Venlo, the Netherlands).

The study was approved by a local ethical committee (CPP Ile-de-France VI, approval n° 60-08, Paris, France, 25 June 2008), the “Commission Informatique et Libertés” (CNIL) (approval n° 908359, Paris, France, 14 October 2008) and the French Ministry of Health (ID-RCB: 2008-A00568-47, Paris, France) and was registered on the Clinical Trial Gov website (protocol ID: 2008-01).

#### 2.1.3. Control Individuals

We defined two independent samples of controls with comparable sample sizes and age and sex distribution for the discovery and replication of case control analyses using a random sampling function (SPSS 16) on the SU.VI.MAX cohort (Table 1). SU.VI.MAX is a national sample of middle-age (women, aged 35–60, or men, aged 45–60 years) healthy volunteers living in France and enrolled between 1996 and 2001 in a randomized, placebo-controlled trial testing the benefit of anti-oxidant nutrients on the incidence of cancers and cardiovascular diseases [19]. We included 1635 individuals, aged 35–65 years at baseline who had genotyping available for the eight TagSNPs of *MMP2*. Cardiac echography was not performed in this population and the MVP status is unknown for all participants.

### 2.2. Sequencing of the MMP2 Gene

Genomic DNA was extracted from whole blood using a standard saline protocol. *MMP2* maps on chromosome 16q12.2 and extends on 47.72 kilobases (kb). The gene has 13 exons and encodes three transcripts of 27.72 kb, 25.11 kb, and 24.99 kb. Fifteen pairs of primers were necessary to amplify the whole coding sequences and the intron-exon boundaries (Table S1). Twelve of the 13 exons constituting the coding regions of the *MMP2* gene were successfully sequenced in 47 index cases of families affected by mitral valve prolapse. Genomic DNA from 47 familial cases and four DNAs of unaffected probands were amplified by PCR. Reactions were performed in 25 µL volumes containing 50 ng genomic DNA, 1X reaction buffer (Sigma-Aldrich, Saint-Louis, MO, USA), 1.5 mM MgCl_2_ (Sigma-Aldrich), 0.2 mM each dNTP (Invitrogen, Thermo Fisher Scientific, Waltham, MA, USA), 0.25 µM each of both primers and 1.25 U Taq DNA polymerase (Sigma-Aldrich). The mix was then subjected to 30 cycles of PCR amplification under annealing temperature adapted for each primer with a C1000 thermal cycler (BioRad, Hercules, CA, USA). Primer excess was removed from the amplified fragments with the use of antarctic phosphatase and exonuclease I (Ozyme, St Quentin en Yvelines, France). Purified PCR products were then sequenced with a big dye terminator cycle-sequencing system (Applied Biosystems, Thermo Fisher Scientific) and amplified DNA fragments were separated using an ABI Prism 3730 DNA Analyzer Sequencer (Applied Biosystems, Thermo Fisher Scientific).

### 2.3. SNPs Selection and Genotyping

**Figure 1:**
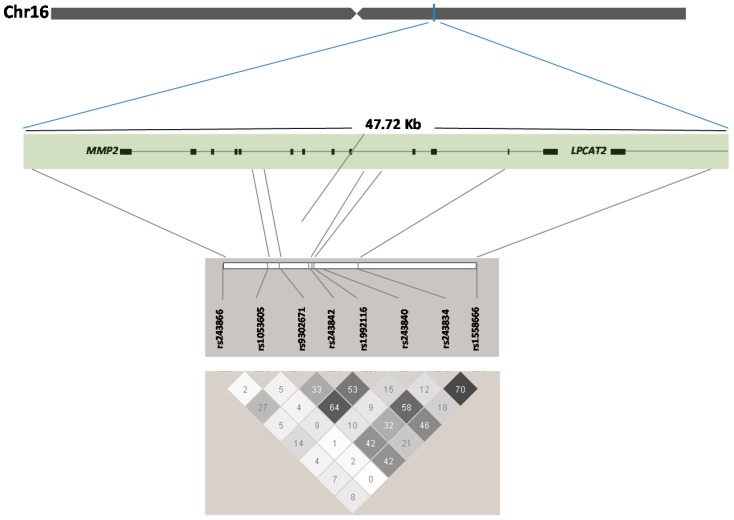
Structure of *MMP2* gene on chromosome 16, position of 8 polymorphisms across the *MMP2* locus and linkage disequilibrium (*r*^2^) between SNPs.

We extracted genotyping data at the *MMP2* locus for the HapMap population of Europeans (CEU, accessed May 2010, Build NCBI 35, Hg 17) and performed a linkage disequilibrium (LD) analysis and identified eight common TagSNPs as having a minor allele frequency (MAF) ≥ 0.05 and presenting low LD between each other (Figure 1). TagSNPs were genotyped in 625 MVP-France Set 1 and 47 familial cases. The LGC genomics company performed the genotyping using a homogeneous fluorescent FRET based system (lgcgenomics.com). Genotyping call rate was at least 99% for all SNPs.

At the replication stage, we used genotypes for rs1558666 that we have generated using genotyping arrays (Illumina 660W-Quad in MVP-France and Illumina Human Hap300 and imputation in SU.VI.MAX) for the purposes of an ongoing genome-wide association study.

### 2.4. Statistical Methods

The correlation between SNPs in HapMap individuals was assessed using Haploview [20]. The association with MVP at each stage was performed by logistic regression under the additive model using PLINK software [21]. Adjusted analyses were logistic regressions where sex and age were included as covariates. Dominant and recessive models were tested and did not provide better association than the additive model, and thus were not presented in the tables.

## 3. Results

### 3.1. Genetic Variation of the Coding Regions of MMP2 in Familial MVP

We did not succeed in amplifying the first exon, probably because of its high GC content (72%), and describe here the results for nine different exons where we identified genetic variants (Table S1). Using the DNAs from 47 patients, we identified 15 different SNPs including one indel and seven non-coding SNPs (six SNPs in the UTR3′ and one SNP in the exon-intron junction). Among the eight coding SNPs, seven are synonymous and one variant (rs368282133) is nonsynonymous and rare with no MAF in HapMap. This variant leads to a missense substitution of an Arginine residue into a Histidine residue (Arg222His) and was observed in two heterozygote patients (MAF in our sample is 0.021). This change occurs in a collagen-binding domain and is described as conserved among mammals and chicken. However, the conservation does not stand for other vertebrates where a Lys is reported for *Xenopus Tropicalis* and zebrafish for instance. On the other hand, the Arg222His is predicted as benign, according to two sequence homology—based tools (SIFT: Sort Intolerant from Tolerant [22] and PolyPhen2: Polymorphism Phenotype [23], score Grantham = 29), which allows a bioinformatics-based prediction of the potential impact of this non synonymous variation on the protein function. Given the limited potential functionality of this variant and the absence of additional affected members in the two families where Arg222His was described, the implication of this variation in the MVP was not further continued.

### 3.2. Association Analyses of Common Variants in MMP2 with MVP

Here we analyzed two sets of MVP-France cases with genetically confirmed European background, defined from an ongoing genome-wide association study (GWAS), to test the association of eight common SNPs with MVP risk in French clinical populations. The first sample (Set 1) corresponds to an intermediate recruitment freeze of MVP-France. This sample is 625 patients aged 62.7 ± 13.1 years, among which 67% had surgery, either valve repair or replacement.

#### 3.2.1. Association Analyses of *MMP2* Common Variants with MVP

We analyzed eight TagSNPs with MAF ranging from 0.06 to 0.48 in the control population as summarizing the genetic information of common variants at the *MMP2 locus* (Figure 1, Table S2). The logistic regression analyses indicated a significant association with MVP for rs1558666 (OR = 0.86 95% CI (0.74–0.99), *p*-value = 0.033). MVP-France displayed an important sex ratio discrepancy compared to controls with 30% and 60% females in cases and controls, respectively (Table 1). To account for this putative confounding factor, we performed adjusted logistic regressions including age and sex as covariates for all SNPs (Table 2). The adjusted analyses strengthen the association of rs1558666 (OR_adj_ = 0.79, *p*-value = 0.003) with MVP and indicate an additional significant association for rs930271 (OR = 0.82 95% CI (0.70–0.96), *p*-value = 0.016). However, only rs158666 remains significantly associated with MVP risk after the application of the Bonferroni corrected *p*-value (*p*_Adj_ = 0.006), which takes into account for multiple testing.

**Table 2:** Association of *MMP2* common tagSNPs with mitral valve prolapse.

SNP	Minor Allele	MAF Cases *n* = 672	MAF Controls *n* = 815	OR [IC 95%]	*p*	OR_Adj_ [IC 95%]	*p*_Adj_
rs1053605	T	0.07	0.06	1.23 [0.90–1.68]	0.196	1.22 [0.87–1.70]	0.251
rs1558666	A	0.45	0.49	0.86 [0.74–0.99]	0.033	0.79 [0.68–0.93]	0.003 *
rs1992116	A	0.41	0.43	0.93 [0.80–1.07]	0.291	0.87 [0.75–1.02]	0.080
rs243834	G	0.49	0.48	1.08 [0.93–1.24]	0.317	1.15 [0.99–1.34]	0.070
rs243840	G	0.20	0.19	1.12 [0.93–1.35]	0.220	1.13 [0.93–1.37]	0.237
rs243842	C	0.38	0.39	0.98 [0.85–1.14]	0.832	1.05 [0.90–1.23]	0.540
rs243866	A	0.23	0.24	0.96 [0.81–1.14]	0.637	0.95 [0.79–1.14]	0.584
rs9302671	T	0.33	0.36	0.88 [0.75–1.02]	0.091	0.82 [0.70–0.96]	0.016

OR: Odds ratios. *p*: *p*-value generated by the logistic regression test performed to compare the prevalence of minor alleles in cases and controls under the additive model. OR_Adj_ and *p*_Adj_ indicate ORs and *p*-values from the logistic regression analyses including age and sex are covariates. *: Significant associations after Bonferroni correction, with corrected *p*-value = 0.05/8 = 0.006.

The valve repair or valve replacement occurs when mitral regurgitation is established and patients are considered to be in a more severe stage of the disease. As a surrogate marker of mitral regurgitation, we tested the association of *MMP2* variants by comparing allele frequencies of MVP patients who had surgery (*n* = 421) to the total sample of SU.VI.MAX Set 2 (*n* = 815). All SNPs studied showed comparable effects on MVP risk, with rs1558666 showing a significant association with MVP when adjusted for age and sex but not below the corrected threshold (OR = 0.83, *p*-value = 0.036, Table S2).

#### 3.2.2. Association Analyses of *MMP2* Common Variants Stratified on Myxomatous and Fibroelastic Deficiency MVP

Among the 672 MVP patients that we analyzed (Set 1), 414 patients were diagnosed a myxomatous valve phenotype or Barlow disease while 116 were defined as non-myxomatous valve prolapse. In two out of three patients, the myxomatous phenotype was defined during surgery intervention and was reported in the surgery report. On average, Barlow disease patients were seven years younger at examination and had similar sex ratio and surgery percentage to the non-myxomatous patients (Table 1).

The stratified association analyses show comparable results to the global analyses in term of effects and significance for *MMP2* SNPs except for rs1558666 with the non-myxomatous phenotype (OR_adj_ = 0.67 (0.51–0.89), *p*-value = 0.006, Table 3).

**Table 3:** Association of *MMP2* common tagSNPs with myxomatous (Barlow disease) and non-myxomatous mitral valve prolapse.

SNP	Minor Allele	MAF Cases	MAF Controls	OR [95% CI]	*p*	OR_Adj_ [95% CI]	*p*_Adj_
Myxomatous (Barlow) MVP							
rs1053605	T	0.06	0.06	1.10 [0.77–1.58]	0.599	1.01 [0.68–1.50]	0.968
rs1558666	A	0.46	0.49	0.88 [0.75–1.04]	0.130	0.83 [0.69–0.99]	0.036
rs1992116	A	0.40	0.43	0.91 [0.77–1.07]	0.237	0.84 [0.71–1.01]	0.065
rs243834	G	0.49	0.48	1.08 [0.92–1.27]	0.355	1.18 [0.99–1.41]	0.064
rs243840	G	0.21	0.19	1.16 [0.94–1.42]	0.163	1.17 [0.93–1.47]	0.171
rs243842	C	0.38	0.39	0.99 [0.84-1.16]	0.861	1.06 [0.89–1.27]	0.509
rs243866	A	0.23	0.24	0.93 [0.77–1.13]	0.484	0.96 [0.78–1.19]	0.716
rs9302671	T	0.34	0.36	0.89 [0.75–1.05]	0.170	0.83 [0.68–1.00]	0.049
Non-Myxomatous (FED) MVP							
rs1053605	T	0.08	0.06	1.43 [0.84–2.46]	0.191	1.47 [0.85–2.54]	0.173
rs1558666	A	0.40	0.49	0.71 [0.54-0.93]	0.012	0.67 [0.51–0.89]	0.006 *
rs1992116	A	0.40	0.43	0.89 [0.68–1.18]	0.417	0.87 [0.65–1.15]	0.329
rs243834	G	0.55	0.48	1.33 [1.01–1.75]	0.042	1.37 [1.03–1.82]	0.029
rs243840	G	0.21	0.19	1.19 [0.85–1.67]	0.322	1.17 [0.83–1.66]	0.364
rs243842	C	0.38	0.39	0.99 [0.75–1.30]	0.929	1.02 [0.77–1.35]	0.888
rs243866	A	0.23	0.24	0.92 [0.67–1.27]	0.622	0.91 [0.66–1.26]	0.570
rs9302671	T	0.30	0.36	0.77 [0.57–1.03]	0.080	0.75 [0.55–1.02]	0.063

OR: Odds ratios. *p*: *p*-value associated to the logistic regression test performed to compare the prevalence of minor alleles in cases and controls. OR_Adj_ and *p*_Adj_ indicate ORs and *p*-values from the logistic regression analyses including age and sex are covariates. *: Significant associations after Bonferroni correction, with corrected *p*-value = 0.006.

#### 3.2.3. Validation of rs1558666 as a Genetic Susceptibility Variant for Non-Myxomatous MVP

At the end of the recruitment of the MVP-France protocol, additional patients were available (*n* = 401) with overall similar clinical characteristics to the first sample (Table 1) that we initially analyzed in the *MMP2* genetic investigation. We decided to use this sample of MVP cases to validate the suggestive association observed for rs1558666 and compared the allele frequencies to the SU.VI.MAX Set 2 as controls.

Table 4 summarizes the association of rs1558666 with MVP. In the initial association analyses, we observed a lower MAF of rs1558666 in the cases compared to the controls in all the samples analyzed, with the lowest frequency observed in the non-myxomatous group (MAF = 0.40) and the highest in the controls (MAF = 0.49, Table 4). In the validation analyses, while the MAF in the controls was almost identical (MAF = 0.49 in SU.VI.MAX Set 1 and MAF = 0.48 in SU.VI.MAX Set 2), we obtained higher frequency for the rs1558666 in MVP cases (MAF = 0.51). Thus, the association that we observed initially was not significantly replicated in the additional sample with an opposite effect (OR = 1.14, *p* = 0.169). Accordingly, the global analysis was also non conclusive (OR = 0.91, *p* = 0.126). Interestingly, the stratified analysis in non-myxomatous MVP cases only showed consistent direction of the effect of the rs1558666 in the validation sample, with a MAF lower in the cases (MAF = 0.44) compared to the controls (MAF = 0.48), despite a lack of significance (OR = 0.84, *p* = 0.38) in this rather smaller and underpowered sample of patients (*n* = 57). Consequently, the association with the non myxomatous phenotype of MVP reached significance in the global sample (OR = 0.73, *p* = 0.005, N_Cases_ = 173). 

## 4. Discussion

In our genetic investigation of metalloproteinase 2 gene (*MMP2*) we assessed the role of rare coding variants and discarded their implication in the etiology of familial, mainly early onset forms of MVP and described a suggestive association between rs1558666, a common variant near *MMP2*, and MVP risk in French populations.

In order to optimize the genetic coverage of the *MMP2* locus, we included all common genetic variants representative of the linkage disequilibrium (LD) block of *MMP2*, which also includes *LPCAT2* encoding lysophosphatidylcholine acyltransferase 2, putatively involved in membrane biosynthesis and production of platelet-activating factor in inflammatory cells (Figure 1). LPCAT2 function has no obvious biological link with valve integrity or development. According to our association result, the major allele (G) at rs1558666 is more prevalent in MVP patients compared to control participants. The rs1558666 maps downstream *MMP2*, in a sequence predicted *in silico* to be recognized by C/EBP and in interaction with nuclear proteins because protected in a DNase sequencing experiment in the endothelial cells model HUVEC, as reported by the ENCODE project (regulome.db.org and [24]). According to the recent data from the pilot study of the gene by tissue expression (GTEx) project [25], the MVP risk allele of rs1558666 tends to correlate with higher expression of *MMP2* in heart left ventricle human samples (*p* = 0.07). Because mitral valve tissue is not collected in the GTEx project, further investigation in this specific tissue is needed to confirm the putative effect of rs1558666 on MVP risk by increasing *MMP2* expression.

In support of this hypothesis is the increased *MMP2* activity reported in diseased mitral valves [15,26]. Histological analyses of myxomatous mitral valves show changes in the amount of collagen, elastin and fibrillin, and a disorganized distribution pattern and structural abnormalities of these components. These abnormalities include spiraling collagen fibrils and alterations in the pattern of arrangement of collagen bundles, structural changes in the organization of elastic fibers, an increase in the number and a decrease in size of the elastic fibers [27,28,29]. Myxomatous mitral valves are also characterized by an accumulation of proteoglycans, particularly in the spongiosa layer, and changes in their composition [30,31]. Moreover, the spongiosa contains an increased number of interstitial cells, which have properties of activated myofibroblasts. These cells express raised concentrations of various proteolytic enzymes including matrix metalloproteinases (MMPs) [14]. The physiological function of MMPs is the modulation and the regulation of extracellular matrix (ECM) turnover by direct proteolytic degradation of the ECM proteins, including collagen, fibronectin, and proteoglycans [13].

**Table 4:** Association of rs1558666 with mitral valve prolapse in the initial association studies, the validation and the global analyses.

rs1558666	Initial Associations					Validations					Global Analyses				
	N_Cases_	MAF Cases	MAF Controls	OR_Adj_	*p*_Adj_	N_Cases_	MAF Cases	MAF Controls	OR_Adj_	*p*_Adj_	N_Cases_	MAF Cases	MAF Controls	OR_Adj_	*p*_Adj_
				[95% CI]					[95% CI]					[95% CI]	
All forms	672	0.45	0.49	0.79	0.003	401	0.51	0.48	1.14	0.169	1077	0.47	0.48	0.91	0.126
				[0.68–0.93]					[0.95–1.37]					[0.81–1.03]	
Myxomatous (Barlow)	414	0.46	0.49	0.83	0.036	255	0.52	0.48	1.17	0.165	669	0.48	0.48	0.94	0.414
				[0.69–0.99]					[0.94–1.46]					[0.82–1.08]	
Non Myxmatous (FED)	116	0.40	0.49	0.67	0.006	57	0.44	0.48	0.84	0.385	173	0.41	0.48	0.73	0.005
				[0.51–0.89]					[0.57–1.24]					[0.58–0.91]	
Surgery Cases	421	0.46	0.49	0.83	0.036	322	0.51	0.48	1.16	0.147	743	0.48	0.48	0.96	0.502
				[0.70–0.99]					[0.95–1.41]					[0.84–1.09]	

OR_Adj_ and *p*_Adj_ indicate odds ratios and *p*-values from the logistic regression tests performed to compare the prevalence of minor alleles frequencies (MAF) in cases and controls, including age and sex are covariates. FED: Fibroelastic deficiency.

The overexpression of *MMP2* specifically in the heart of mice resulted in normal mice at ages four to six months [17]. This finding in the mouse is consistent with the lack of causative rare mutations in the more severe and early onset MVP phenotype that characterizes the familial patients. Thus, the overexpression of *MMP2* reported in diseased valves [15] is probably a secondary defect, not due to genetic defaults. Actually, the echocardiographic MVP manifests in transgenic mice at a later age (at 12 to 14 months) [17], which is consistent with the association of a common variant with sporadic forms of MVP more likely to be due to degenerative process overtime. Thus, *MMP2*’s role in the etiology of MVP is putatively a long process of susceptibility, genetic background, and aging and/or mechanical stress interactions. This hypothesis needs confirmation using a prospective genetic epidemiological setting to assess the extent to which the rs1558666 risk allele carriers are more prone to mitral valve degeneration, compared to non-carriers. On the other hand, the existence of rare coding variants that would result in the increase of *MMP2* levels of activity is unlikely. Only regulatory regions are susceptible to harbor such variants and their investigation would be warranted.

## 5. Conclusions

Our genetic investigation of *MMP2* suggests a limited implication of rare coding genetic variants in the inheritance of non-syndromic MVP in French families and suggests the association of common variants near this gene with sporadic MVP in two cases controls studies. More comprehensive genomic investigation such as exome sequencing and genome-wide association studies are more likely to decipher the genetic basis of MVP.

## Supplementary Materials

Supplementary materials can be accessed at: http://www.mdpi.com/2308-3425/2/3/0176/s1.
