# Supplementary material for: Investigation of the Matrix Metalloproteinase-2 Gene in Patients with Non-Syndromic Mitral Valve Prolapse
Source: J Cardiovasc Dev Dis. 2015 Jul 10;2(3):176–89. doi: 10.3390/jcdd2030176 (PMC5753144; doi:10.3390/jcdd2030176)
Supplement: Supplementary file 1 [file jcdd-02-00176-s001.pdf]

Supplementary Materials

Table S1. Primers used to amplify *MMP2* coding region prior to direct sequencing.

MMP2 Exons	Forward Primer	Reverse Primer
1	5′-AGTGGAGGAGGGCGAGTAG-3′	5′-CCCAAGCTGTTTACCGAAG-3′
2	5′-ACCCAGTACTCCACCCAGT-3′	5′-TCACAGCACTGAAAAGTAACC-3′
3	5′-GGCACATGCTCACATATACA-3′	5′-TATTGGACAGCACAGTAGACC-3′
4 and 5	5′-CAGGGTCTAGGTGGCACAG-3′	5′-GTTGGAGGGATGCTCATTAAC-3′
6	5′-ATTGGGATAACCAGGGAAG-3′	5′-CCCAATGTCTGTTCTGATTT-3′
7	5′-GGTGGGTGAGATGAGTCTAA-3′	5′-GGATCTAAGCAGGGACCTT-3′
8	5′-CAGGGTTCTCAGCCTTACT-3′	5′-GGATAGGGAAGAGTTATCCAA-3′
9	5′-GTACAAAGGGCCCCAGGAC-3′	5′-AGAACAACAGGCAGAGCACAC-3′
10	5′-GTAGAGGGAGAGAACAGGGTA-3′	5′-GTGTACACTGAGGGTTAATGC-3′
11	5′-CTGTGTGAGCCCTGATTCT-3′	5′-ACCTTTGGCAACATCTCAG-3′
12	5′-AGATTGTTTTTATGGGGTCAT-3′	5′-GTGAGAAGTCTTTCTGGGTCT-3′
13	5′-CCAGGCAGAAATTCAAAGT-3′	5′-AGCAAACTGCTAATGTAAGAAAG-3′
13	5′-CTGTCTCAAGAGGGCACTG-3′	5′-CAACAAAATTGCTGATTCTTC-3′
13	5′-GTGTCTGCTGGAAAGGTCAGA-3′	5′-GTGGTGGGGGTGTTTTTGA-3′

Table S2. List of identified SNPs by direct sequencing in *MMP2*.

MMP2 Exon	Alleles	Chr: bp (hg17)	Type	ID dbSNP	MAF	MAF CEU
4 and 5	*G/A*	16: 54077023	NON_SYNONYMOUS_CODING	rs368282133 *	0.021	NA
4 and 5	G/C	16: 54077036	SYNONYMOUS_CODING	rs1132896	0.298	0.427
4 and 5	C/T	16: 54077108	SYNONYMOUS_CODING	rs1053605	0.006	0.075
6	*C/T*	16 : 54079978	SYNONYMOUS_CODING	NA	0.011	NA
7	T/C	16: 54081206	SYNONYMOUS_CODING	rs243849	0.830	0.883
9	G/A	16: 54084614	SYNONYMOUS_CODING	rs2287074	0.340	0.500
12	A/G	16: 54094188	SPLICE_SITE, INTRONIC	rs243834	0.544	0.467
12	C/T	16: 54094228	SYNONYMOUS_CODING	rs14070	0.372	0.337
12	C/G	16: 54094264	SYNONYMOUS_CODING	rs11541998	0.128	0.108
13	T/C	16: 54097014	3′UTR	rs17860019	0.021	0.033
13	-/T	16: 54097021-54097020	3′UTR	rs36115725	0.106	NA
13	G/A	16: 54097025	3′UTR	rs41280909	0.021	0.005
13	A/G	16: 54097038	3′UTR	rs111371964	0.021	0.005
13	A/C	16: 54097115	3′UTR	rs7201	0.372	0.491
13	C/T	16: 54097727	3′UTR	rs140455191	0.021	0.005

Minor allele frequencies (MAF) are allele frequencies observed in the 47 MVP affected patients and were compared to the frequencies described in European descent individuals from the CEU population from the Hapmap project or the 1000 genomes project. Positions on Chr16 are indicated according to build hg17. * This variant corresponds to the missense variants Arg222His observed in 2 MVP patients.

Table S3. Association of *MMP2* common tagSNPs with MVP in patients who underwent valve surgery repair.

SNP	Minor Allele	MAF Cases	MAF Controls	OR [95% CI]	*p*	OR_{Adj} [95% CI]	p_{Adj}
rs1053605	T	0.06	0.06	1.19 [0.84–1.70]	0.327	1.26 [0.86–1.84]	0.242
rs1558666	A	0.46	0.49	0.89 [0.76–1.05]	0.156	0.83 [0.70–0.99]	0.036
rs1992116	A	0.42	0.43	0.95 [0.80–1.12]	0.537	0.91 [0.76–1.09]	0.311
rs243834	G	0.50	0.48	1.09 [0.92–1.28]	0.316	1.15 [0.96–1.37]	0.132
rs243840	G	0.20	0.19	1.12 [0.91–1.38]	0.273	1.10 [0.88–1.38]	0.400
rs243842	C	0.38	0.39	0.96 [0.82–1.13]	0.639	1.02 [0.85–1.22]	0.839
rs243866	A	0.24	0.24	1.02 [0.84–1.23]	0.877	1.01 [0.82–1.25]	0.911
rs9302671	T	0.34	0.36	0.91 [0.76–1.08]	0.271	0.85 [0.71–1.03]	0.091

MAF: minor allele frequency. OR: odds ratios. *p*: *p*-value associated to the logistic regression test performed to compare the prevalence of minor alleles in cases and controls. OR_{Adj} and p_{Adj} indicate ORs and *p*-values from the logistic regression analyses including age and sex are covariates.
